# Student preparedness characteristics important for clinical learning: perspectives of supervisors from medicine, pharmacy and nursing

**DOI:** 10.1186/s12909-017-0966-4

**Published:** 2017-08-08

**Authors:** Hasini Banneheke, Vishna Devi Nadarajah, Srinivasan Ramamurthy, Afshan Sumera, Sneha Ravindranath, Kamalan Jeevaratnam, Benny Efendie, Leela Chellamuthu, Purushotham Krishnappa, Ray Peterson

**Affiliations:** 1 Faculty of Medical Sciences, University of Sri Jayewardenepura, Colombo, Sri Lanka; 2 School of Medicine, International Medical University, No 126 Jalan Jalil Perkasa 19, Bukit Jalil, 57000 Kuala Lumpur, Malaysia; 3 School of Pharmacy, International Medical University, Kuala Lumpur, Malaysia; 4 School of Dentistry, International Medical University, Kuala Lumpur, Malaysia; 5 School of Medicine, Perdana University, Selangor, Malaysia; 6 School of Pharmacy, Monash University, Selangor, Malaysia; 7 School of Health Sciences, International Medical University, Kuala Lumpur, Malaysia; 8 Faculty of Health Sciences, Adelaide Medical School, University of Adelaide, Adelaide, Australia

**Keywords:** Clinical preparedness, Supervisor’s perspective, Clinical learning

## Abstract

**Background:**

Student perspectives of clinical preparedness have been studied in the literature, but the viewpoint of supervisors is limited. Hence, the aim was to examine the perspective of supervisors on the characteristics of health professional students important for preparedness for clinical learning.

**Methods:**

This was a descriptive, questionnaire-based, cross-sectional study conducted at three higher education institutions in Malaysia. A previously published questionnaire with 62 characteristics was adopted with modifications after pre-testing. Descriptive analysis was completed for the demographic data. The sample was grouped based on health profession, clinical practice experience and teaching experience for further analysis. Non-parametric Kruskal-Wallis test was selected to evaluate differences in mean ranks to assess the null hypothesis that the medians are equal across the groups. Kruskal-Wallis post-hoc pair wise comparison was performed on samples with significant differences across samples.

**Results:**

The sample was comprised of 173 supervisors from medicine (55, 32%), pharmacy (84, 48%) and nursing (34, 20%). The majority (63%) of the supervisors were currently in professional practice. A high percentage (40%) of supervisors had less than 4 years of teaching experience. The highest theme ratings were for willingness (6.00) and professionalism (5.90). There was a significant difference (*p* < 0.05) in the medians, among medicine, pharmacy and nursing professional speciality for willingness (5.70, 6.00 and 6.00), professionalism (5.70, 5.90 and 6.15), communication and interaction (5.42, 5.67 and 6.00), personal attributes (5.42, 5.71 and 6.02) and the professional and interpersonal skills (5.50, 5.63 and 6.00) themes. Post-hoc analysis showed a significant difference (*p* < 0.05) between medicine and nursing groups in the willingness (5.70 and 6.00), professionalism (5.70 and 6.15) and personal attributes (5.42 and 6.02) themes. Supervisors who are currently in practice had given high ratings compared to other groups. There were no significant differences observed within groups with different level of teaching experiences.

**Conclusions:**

All supervisors rated professionalism and willingness as the most important characteristics followed by personal attributes. Further strengthening learning opportunities related to these characteristics in the curriculum may improve the students’ preparedness in clinical learning.

**Electronic supplementary material:**

The online version of this article (doi:10.1186/s12909-017-0966-4) contains supplementary material, which is available to authorized users.

## Background

Medicine, pharmacy, and nursing students gain subject-specific knowledge during the initial part of their undergraduate study mainly through plenaries, small group learning, training in clinical skills or laboratories with occasional visits to hospitals and community centres. Practice related contextual learning that takes place later in their training helps students understand the course content better, integrate and apply the knowledge for patient-centred care, inculcate correct attitudes and gain skills through practice [1]. Through this process, the novice undergraduate student transforms into a work-ready health care practitioner. Nevertheless, the transition from the pre-clinical phase of study into the clinical learning environment remains stressful for the students [2–5] and they can suffer from initial clinical anxiety [6, 7] due to several reasons; (i) the differences in learning environments and teaching styles, (ii) the theory-practice gap, (iii) issues in working relationship with either health care practitioners, peers or patients, (iv) the fear of making mistakes, (v) apprehension over clinical educators’ evaluations, (vi) ambiguity of their role and responsibilities in a healthcare setting, and (vii) workload and performance expectations [2, 7–9]. Efforts are undertaken to increase student clinical preparedness [10] and reduce the stress for students during the transition by modifying the curriculum. Curricula modifications include incorporation of clinical skills and simulation sessions from year 1, the appointment of peer instructors and mentors [11], provision of short transitional courses [12] and clinically-related resource materials [13, 14], access to clinical teachers [14], increasing hospital visits, general practice postings and community projects starting early and in the preclinical phase. However, even after incorporating these measures, evidence suggest that students are not at ease but continue to struggle during transition [15, 16]. Although preparedness of students is important, the difficulty in accessing clinical learning environments, priority for patient care and safety, availability of human resources and the high costs of training should also be considered [17, 18].

Student perspectives of clinical preparedness and transition have been studied in the literature [4, 19–23]. However, the viewpoint of supervisors from various health professional courses who direct the students to gain professionally related skills in the clinical environment is limited [10, 24]. Researchers have obtained student viewpoints in relation to learning environments, intellectual climate, and the relationship with fellow students and supervisors [25, 26]. Students have reported that interest in the subject, a meaningful learning environment and a positive student-supervisor relationship are the most important aspects for effective clinical learning. Supervision is an essential component in work based contextual learning for clinical students. Information from the existing literature has helped curriculum planners to optimize their programmes. However, clinical settings and expectation are contextually different and will change over time and needs updating [27].

In this study, we considered the supervisors’ perspectives on the characteristics of health professional students that are important for preparedness for clinical learning. Given that students from various health professions such as medicine, nursing and pharmacy share similar clinical learning contexts, it is valuable to obtain the perspectives of the supervisors in these health professions. The data assists by exploring any differences in perceptions based on clinical practice experience, health profession specialty and teaching experience. A recent paper by Chipchase et al.*,* describes the supervisors’ views on students’ clinical preparedness from disciplines such as occupational therapy, physiotherapy, and speech pathology [24] and identifies six themes that can be used as indicators for student preparedness for the clinical learning environment. The six themes are knowledge and understanding, willingness, professionalism, communication and interaction, personal attributes, and professional and interpersonal skills. We planned our study based on the questionnaire of Chipchase et al., so that we can explore these themes and characteristics in the context of other health professionals. In addition, there are no comparative studies in the literature expressing the views of supervisors from medicine, pharmacy, and nursing on student clinical preparedness. The hypothesis is that there are differences between medicine, pharmacy and nursing supervisors on the characteristic that are important for students preparedness for clinical learning. Any differences or similarities in views would be useful for planning clinical teaching and learning in a resource efficient manner. Being multi-professional and healthcare-based, the findings of our study are applicable to any medical or health sciences school.

## Methods

The aim was to examine medicine, pharmacy and nursing supervisors’ perspectives on the characteristics of health professional students that are important for preparedness for clinical learning.

### Study design, setting and participants

This was a descriptive, questionnaire-based, cross-sectional study. It was conducted at three health sciences institutions in Kuala Lumpur, Malaysia, namely; International Medical University [IMU] (medical (60), pharmacy (77) and nursing (34) supervisors), Perdana University (medical supervisors only (15)) and Monash University, Malaysia (pharmacy supervisors only (39)). All supervisors from the medical, pharmacy and nursing professions who were involved in preparing students for clinical learning were invited and consented to participate. Data collection was carried out over a period of 4 months.

### Study instrument and data collection

The questionnaire with 62 characteristics developed by Chipchase et al., 2012 [24] was adopted. These 62 items are organized into six themes, namely knowledge and understanding, willingness, professionalism, communication and interaction, personal attributes, and professional and interpersonal skills. In addition to the 62 items, we added a section for demographic data and two open-ended questions for suggestions and comments. The copy of the modified Chipchase et al. questionnaire is attached in Additional file 1. The language of the survey was in English. A pilot study was carried out to validate the modified questionnaire on a sub-sample (*n* = 20) of IMU supervisors. Those 20 participants involved in the pilot study were excluded from the main study. The six themes in the original questionnaire were removed at the time of data collection to reduce any potential bias in responding to each item. However, the 62 items were grouped under six themes at the time of data analysis. Item responses were based on a seven-point Likert scale with 1 = not important, 2 = slightly important, 3 = somewhat important, 4 = moderately important, 5 = important, 6 = very important and 7 = extremely important.

### Data analysis

Data entry and statistical analysis were completed using IBM SPSS Statistics for Windows, Version 20.0 (IBM Corp. Armonk, New York). Demographic data were analysed using descriptive analysis. Medians of the seven-point Likert scale for all 62 items were calculated. The open comments were categorized into the six themes in the original questionnaire independently by two researchers. We grouped the supervisors based on their health profession (medicine, pharmacy and nursing). In addition, we investigated how their clinical practice experience and their years of teaching experience changes their perception on the characteristic important for clinical learning. The supervisor groups were further subdivided based on clinical practice experience for each health profession (currently in practice [group-1], practiced in the past but not at present [group-2] and only practiced during study course/training [group-3]), and teaching experience for each health profession (less than 4 years [group-A], 5–9 years [group-B] and more than 10 years [group-C]). These subdivisions were selected to better understand the perceptions of supervisors on the characteristic important for clinical learning in the medicine, pharmacy and nursing professions.

All results were subjected to normality testing. Based on the Shapiro-Wilks normality test results, the non-parametric Kruskal-Wallis test was selected to evaluate differences in mean ranks and to assess the null hypothesis that the medians were equal across the groups. A Kruskal-Wallis post-hoc pairwise comparison was performed on samples, which showed significant difference across samples.

## Results

A total of 173 supervisors from medicine, pharmacy, and nursing participated in the study. The participation from each institution was as follows; International Medical University (119), Monash University (39) and Perdana University (15). The clinical supervisors from the three institutions consisted of nationalities from Malaysia, Myanmar, India, Pakistan, Sri Lanka, Australia, The United Kingdom, Ireland, Canada, The United States and the Middle Eastern region. The characteristics of supervisors is shown in Table 1. In total, there were more female participants (61%) than males. In terms of clinical experience in their own health profession, the majority (62%) of the supervisors reported that they are currently in practice, while 23% had practiced in the past, and 15% have no clinical work experience. The majority of those with no clinical work experience were pharmacists. A slightly higher percentage (40%) of supervisors had less than 4 years of teaching experience.

**Table 1:** Characteristics of supervisors for medicine, pharmacy and nursing

Characteristics of supervisors	Medicine *N* = 55 (%) (32)	Pharmacy *N* = 84 (%) (49)	Nursing *N* = 34 (%) (19)	Total Surveyed *N* = 173 (%)
Gender				
Male	32 (58)	32 (38)	4 (12)	68 (39)
Female	23 (42)	52 (62)	30 (88)	105 (61)
Clinical practice experience (in years)^a^				
Currently in practice (group-1)	28 (51)	46 (55)	34 (100)	108 (62)
Practiced in the past but not at present (group-2)	27 (49)	12 (14)	0 (0)	39 (23)
Only during study course/training(group-3)	0 (0)	26 (31)	0 (0)	26 (15)
Years involved in clinical teaching^a^				
Less than 4 years (group-A)	11 (20)	49 (58)	9 (26)	69 (40)
5–9 years (group-B)	15 (27)	28 (33)	5 (15)	48 (28)
More than 10 years (group-C)	29 (53)	7 (08)	20 (59)	56 (32)

^a^There were non-respondents

Number (*N*=) and percentage (%)

### Supervisors’ ratings related to characteristics of health professional students important for better preparedness for clinical learning

All supervisors’ scores were above five (5) for all themes related to characteristics of health professional students which were important for better preparedness for clinical learning (Table 2). The median rating of the total sample was highest for the theme willingness (6.00) followed by professionalism (5.90), personal attributes (5.70), communication and interaction (5.66), professional and interpersonal skills (5.50), and knowledge and comprehension (5.36).

**Table 2:** Professional Speciality: Medicine, pharmacy and nursing supervisors’ ratings (median value) for each theme related to characteristics of health professional students important for better preparedness for clinical learning

Theme	Medicine	Pharmacy	Nursing	Overall rating
Knowledge and understanding	5.32	5.63	5.95	5.36
Willingness	5.70*	6.00	6.00*	6.00
Professionalism	5.70**	5.90	6.15**	5.90
Communication and interaction	5.42	5.67	6.00	5.66
Personal attributes	5.42*	5.71	6.02*	5.70
Professional and interpersonal skills	5.50	5.63	6.00	5.50

*p < 0.05, ** *p* < 0.01 for comparison between Medicine and Nursing

### Supervisors’ ratings based on professional specialty

There were three categories of health professions analysed, namely medicine, pharmacy, and nursing. The median values for the six themes for each speciality are reported in Table 2.

The Shapiro-Wilk test for normality showed significant differences (*p < 0.05)* for all themes. It also confirmed that the distribution was not normal.

The non-parametric Kruskal-Wallis test showed that there was a significant difference ((*p < 0.05)* among medicine, pharmacy, and nursing professionals in the median gauging the level of importance of themes as follows: willingness χ2 (2, *n* = 171) = 9.699, *p = 0.008*; χ2 (2, *n* = 171) = 8.956, *p = 0.011* for professionalism; χ2 (2, *n* = 171) = 7.788, *p = 0.020* for personal attributes and χ2 (2, *n* = 171) = 6.235, *p = 0.044* for communication and interaction. With regard to knowledge and professional and interpersonal skills, no significant differences in the medians among the three professional specialties were observed.

Kruskal-Wallis post-hoc pairwise comparisons demonstrated a significant difference (*p < 0.05)* between medicine and nursing groups in willingness, professionalism, and personal attributes. The analysis between the medicine and pharmacy groups and the pharmacy and nursing groups showed no significant difference for all six themes.

### Supervisors’ ratings based on clinical practice experience

Each professional speciality was subcategorised into 3 groups based on clinical practice experience (ie Group-1: currently in practice; Group-2: practiced in the past but not at present; Group-3: practice experience only during study course/training). Only in the pharmacy profession were there participants in all three levels of clinical experience (groups 1, 2 and 3). In medicine, only two levels of clinical experience (group 1 and 2) were present, and in nursing, all the participants belonged to group 1(i.e. currently in practice). The median values for the six themes for the pharmacy and medicine professions are shown in Table 3.

**Table 3:** Clinical Practice Experience: Medicine and pharmacy supervisors’ ratings (median value)

Theme	Pharmacy			Medicine	
	Currently in practice (group-1)	Practiced in the past but not at present (group-2)	Only during study course/training (group-3)	Currently in practice (group-1)	Practiced in the past but not at present (group-2)
Knowledge and understanding	5.60	5.36	5.10	5.36	5.22
Willingness	6.00	6.05	5.80	5.85	5.60
Professionalism	6.00	5.95	5.80	5.80	5.60
Communication and interaction	5.67	5.50	5.67	5.50	5.33
Personal attributes	5.82	5.62	5.47	5.45	5.41
Professional and interpersonal skills	5.63*	5.31	5.00*	5.50	5.44

*p < 0.05 for comparison between currently in practice group (group-1) and only during study course/training (group-3)

In medicine, no significant difference was observed between groups 1 and 2 for all the six themes. For the pharmacy profession, there were significant differences (*p < 0.05)* in all the six themes as observed from the Shapiro-Wilk normality test. It also showed that the population was not normally distributed. Furthermore, the non-parametric Kruskal-Wallis test indicated that there was a significant difference χ2 (2, *N* = 83) = 6.342, *p = 0.042* among three groups in the professional and interpersonal skills theme, while there was no significant difference observed for the other 5 themes. The post-hoc pairwise comparison indicated that there was a significant difference (*p < 0.05)* in the professional and interpersonal skills themes between group 1 and 3. There was no significant difference observed between group 1 and 2 and group 2 and 3.

### Supervisors’ ratings based on teaching experience

Each professional speciality was subcategorised for analysis based on the years of teaching experience into 3 groups categorized as Group-A: less than 4 years, Group-B: 5–9 years and Group-C: more than 10 years. The median values for the six themes for pharmacy, medicine, and nursing professions are shown in Table 4.

**Table 4:** Years involved in clinical teaching: Medicine, pharmacy and nursing supervisors’ ratings (median value)

Theme	Pharmacy			Medicine			Nursing		
	Less than 4 years (group-A)	5–9 years (group-B)	More than 10 years (group-C)	Less than 4 years (group-A)	5–9 years (group-B)	More than 10 years (group-C)	Less than 4 years (group-A)	5–9 years (group-B)	More than 10 years (group-C)
Knowledge and understanding	5.28	5.38	5.90	5.18	5.59	5.75	5.18	6.00	6.00
Willingness	6.00	6.00	6.20	5.70	5.59	5.50	6.00	6.50	6.00
Professionalism	5.80	6.15	6.00	5.70	5.55	5.90	6.00	6.40	6.20
Communication and interaction	5.58	5.50	6.00	5.33	5.58	5.33	6.00	5.67	6.00
Personal attributes	5.71	5.76	5.65	5.24	5.85	5.29	5.29	6.35	6.26
Professional and interpersonal skills	5.56	5.38	5.75	5.25	5.50	5.25	5.00	6.62	6.31

For the three professions, the three groups were subjected to a normality test followed by the non-parametric Kruskal-Wallis test. There were no significant differences observed within three groups for the three professions indicating that the years of teaching experience has no impact on supervisors ratings.

### Supervisors’ responses to open-ended questions

We invited the supervisors to state any other student attributes that are important for clinical learning that had not been mentioned or covered in the questionnaire. Twenty-one percent (*n* = 36) of the participants had responded. The summary of the salient suggestions that were matched with the themes are reported in Table 5.

**Table 5:** Summary of other characteristics suggested by the participants as important for clinical learning

Characteristic	Theme
The student recognizes that he/she is in a privileged position regarding access to/interaction with patients.	Knowledge and understanding
The student understands the need for and is committed to life-long learning.	Knowledge and understanding
The student should be aware of the non-pathological aspects of diseases such as its social and mental health impact on the patient and family.	Knowledge and understanding
The student is able to learn to hide their emotions and display professional behaviour even in distressed situations	Professionalism
The student has good patience to listen to the patient’s problem and their interpretation of the disease or symptoms.	Communication and interaction
The student demonstrates capability to adapt to the environmental and social changes in the clinical setting within a short period.	Personal attributes
The student has passion for the subject learning and passion to become a health professional.	Personal attributes
The student has leadership skills.	Personal attributes
The student is able to improve on strengths and correct weaknesses.	Professional and interpersonal skills
The student is able to prioritize their personal agenda related to professional learning and balance the family matters.	Professional and interpersonal skills

There were 8% (*n* = 14) participants who had written comments under the open comments section. The participants suggested combining some characteristics as they felt there were repetition and overlap between characteristics for the same outcome. They also suggested that the Likert scale could have been a 3 points scale. They found it difficult to differentiate responses to the exact point in a 7-point scale.

## Discussion

This study investigated the perception of health professionals from medicine, pharmacy, and nursing on the characteristics important for the preparedness of undergraduate students for clinical training. Although entirely conducted in Malaysia, participants in this study had come from different countries with different training backgrounds. Additionally, there was a fair gender representation of clinical educators, and with experience levels ranging from junior, mid and senior levels for teaching and clinical practice.

The findings demonstrate that all of the themes and associated characteristics outlined in the questionnaire are important for student preparedness for clinical learning. The characteristics outlined in the present study were derived from previous work prepared on a consensus-based approach using the Delphi technique [24]. In that study, it was observed that despite situational differences in the teaching and learning process and professional category, supervisors had similar expectations. Such an observation has been reported previously in another study conducted in Saudi Arabia [28].

The participants in the present study rated professionalism, willingness and personal attributes highly and a similar observation was made in studies from other health sciences programs such as occupational therapy, physiotherapy, speech pathology [24] and nursing [28]. The professionalism theme is defined as professional skills and behaviours in this current study. Professionalism is built on fundamentals such as knowledge, clinical competencies, communication, ethics and legal understandings [29] and it directly influences the clinical learning process and students’ preparedness for clinical learning [30]. Some authors assert that professionalism in a medical career is stage-specific and context related because professional behavior is greatly influenced by personal value, organizational hierarchy and social desirability while others suggest it applies throughout the career [29]. Additionally, the increasing opportunities for commercial gains in clinical medicine and its possible negative impact on patient care have led to a greater expectation of professional responsibility from students from the first day of clinical encounters [30]. Knowledge and understanding and professional and interpersonal skills had received the least rating of importance from supervisors. This may be justified as knowledge and even interpersonal skills could be taught or congregated as they go along in the training [21]. In another study describing ‘successful’ and ‘unsuccessful’ clinical nursing learning, students characteristics such as critical thinking, communication skills, personal attributes such as positivity, eagerness to learn and traits demonstrating willingness such as acceptance of feedback have been highlighted as important and it concludes that unprepared students become unsuccessful professionals later [10].

The nursing supervisors had notably different perceptions for clinical preparedness compared to the medical and pharmacy professionals with higher scores on all six themes. For nursing supervisors, student characteristics related to the themes knowledge and understanding, personal attributes and professional and interpersonal skills were highly significant. This difference in perception may arise from the role nurses’ play within a healthcare setting compared to doctors or pharmacists. Nurses have high, continuous and recurrent patient contact especially in a ward setting collecting medical histories, distributing medications, monitoring patients’ conditions [31], providing health education and information. In addition, nurses often collaborate and communicate with other healthcare professionals. Barriers such as lack of interpersonal skills and mutual respect, poor attitudes toward team working, status differences and communication gaps were more commonly reported by nurses than by doctors as challenges that affect nurses’ and doctors’ working relationships [32, 33]. On the other hand, the contextual and experiential learning for a nursing student starts from year 1. Thus, a nursing professional is developed on the job with much of the learning through feedback from clinical preceptors. This may explain why the nursing supervisors have rated personal attributes and professional and interpersonal skills as more important for clinical preparedness of their students. Meanwhile, the training and practice of medicine and pharmacy have many similarities. For example, the two programmes emphasise the knowledge of basic sciences in the early years of the undergraduate programme with gradual accentuation of clinical learning towards later years.

Clinical supervisors currently in practice assert higher importance on professional and interpersonal skills compared to those with no clinical practice experience. Professional and interpersonal skills encompass skills such as time management, organizational, verbal and written communication, observational, research, social and problem-solving skills. Developing the interpersonal skills of health professionals is imperative in maintaining good clinical standards and will be rewarding for them in the long run. Practising supervisors or preceptors know from their daily experience that both patients and doctors are at risk with poor interpersonal skills [34]. For all three professions, active observation is necessary for the supervisors or preceptors to assess and give feedback to students in training. If these supervisors or preceptors are in practice they are considered more effective and are better role models for clinical learning [35]. However, they have to balance academic responsibilities and patient care within their duty hours [36] and thus they prefer a student with the right personal attributes such as enthusiasm, initiative and a desire to learn.

A study that explored the importance of professional attributes for preparedness for nursing students reported that educators with more years of experience recognized communication and interaction as a vital characteristic for better clinical performance [28]. No difference in this perspective of the supervisors based on the years of teaching experience was identified in this present study.

Awareness of interprofessional learning is an important graduate attribute. As stated in the literature, across the professions, the outcome/competency statements and relevant assessment tools tend to measure interprofessional engagement indirectly under professional communication and professional behaviour descriptors [24]. In our current study, the questionnaire consists of questions such as ‘the student is willing to work as a team with peers, colleagues and other health professionals’ and ‘the student is able to communicate professionally with members of the multidisciplinary team’ which were designed to measure interprofessional engagement. Clinical supervisors from all the three discipline rated these questions highly clearly reflecting that they value the importance of the interprofessional learning (IPL) component. Hence, the further strengthening of IPL components will improve student preparedness for their clinical learning.

The supervisors open comments provided a valuable insight into the characteristics important for student clinical preparedness. Those descriptions were mapped to the clinical preparedness themes and explained why the themes were rated as important or highly important. Curriculum planners should be aware of the expectations of different groups of clinical supervisors. They should provide evidence of appropriate student selection methods, teaching learning activities and assessment tools measuring professionalism, willingness, and affirmative personal attributes. For example, the multiple mini interviews used for selection of students have been shown to be a useful tool to assess non-cognitive attributes of medical and other health professions students [37]. In this study, the scores obtained for each theme identify the priority areas from the supervisor’s perspective. In outcomes/competencies based education, these prioritized attributes could be emphasized or built upon in the curriculum and be provided to students to aid their preparation for clinical learning. This approach will reduce the gap between students and clinical supervisor’s expectations in their clinical learning sites.Similarly, curriculum planners and reviewers should inform students of important characteristics as described in the six themes and ensure that they get the opportunity to learn and demonstrate these characteristics from the beginning of the course and at levels contextual to programme outcomes. It will also be helpful to include an orientation course immediately prior to the clinical transition of students focusing on these characteristics to reduce the levels of stress and anxiety among students. However, an essential action would be to continue giving assistance to students during their clinical learning, whilst being vigilant and then supporting those students who may be struggling to meet the expectations.

The questionnaire used in this study had an exhaustive list of characteristics important for student preparedness for clinical learning. As such, it had resulted in the development of a lengthy questionnaire that became a limitation in the present study as it led to some clinical supervisors declining participation. Further development of the questionnaire with a shorter version would have been beneficial with increased response rates. Although the study involved multi-professional categories, it was not completely multi-centred as only in one institution was there representation of all the professions. As such, this study will enhance the existing literature while providing helpful tips to institutions that face similar issues. It will also model a process that other institutions may follow to identify their contextualized learner expectations.

## Conclusions

All supervisors had rated professionalism and willingness as the two most important themes of characteristics to inculcate prior to clinical training followed by personal attributes**.** There was a significant difference in the perspective of nursing supervisors regarding characteristics important for students’ clinical preparedness compared to medical and pharmacy professionals. In pharmacy, clinical supervisors currently in practice placed a higher importance on professional and interpersonal skills and personal attributes compared to those with no clinical practice experience. There was no difference in the perspective of the supervisors based on the years of teaching experience.

Considering the current interest in inter-professional education and teamwork, it will be interesting to carry out a qualitative study to determine the reasons for the observed differences in expectations among supervisors from different disciplines. Furthermore, we have completed a study among students to determine their perspective regarding characteristics important for clinical learning and results of that study will be published in future.

## Additional file

Additional file 1: Questionnaire on student preparedness for clinical learning-Supervisors’ perspective. (DOCX 26 kb)
