# Supplementary material for: Student preparedness characteristics important for clinical learning: perspectives of supervisors from medicine, pharmacy and nursing
Source: BMC Med Educ. 2017 Aug 8;17:130. doi: 10.1186/s12909-017-0966-4 (PMC5549327; doi:10.1186/s12909-017-0966-4)
Supplement: Additional file 1: — Questionnaire on student preparedness for clinical learning-Supervisors’ perspective. (DOCX 26 kb) [file 12909_2017_966_MOESM1_ESM.docx]

**Questionnaire on student preparedness for clinical learning-Supervisors’ perspective**

**Section-I (Demographic details)**

***Please tick one box***

| 1. Gender | | |  | | | |  |  |  |  |
| --- | --- | --- | --- | --- | --- | --- | --- | --- | --- | --- |
| 🞏 Male | | | | 🞏 Female | |  |  |  |  |  |
| 1. Years involved in Clinical education | | | | | | | | | |  |
|  | 🞏 | 1-4 years | | 🞏 | 5-9 years | | | 🞏 | More than 10 years | |

1. Professional carrier specialty (please select the most appropriate)

🞏 Medicine 🞏 Nursing 🞏 Pharmacy

1. Do you have clinical practice/experience in your field? 🞏 Yes; currently in practice

🞏 Yes; practiced in the past but not at present

🞏 Yes; only during study course/training

**Section-II**

Please respond to each statement below that best reflects how you feel about the **importance of these factors for the preparedness of health profession students’ for clinical learning** when they enter to clinical training from pre clinical phase.

Circle only **ONE** response for each statement.

|  |  | **not important** | **slightly important** | **somewhat important** | **moderately important** | **important** | **very important** | **extremely  important** |
| --- | --- | --- | --- | --- | --- | --- | --- | --- |
| 1. | The student demonstrates sound theoretical knowledge in basic sciences | 1 | 2 | 3 | 4 | 5 | 6 | 7 |
| 2. | The student demonstrates a thorough knowledge of therapy practices relevant to the area | 1 | 2 | 3 | 4 | 5 | 6 | 7 |
| 3. | The student knows how to access information when a gap in knowledge or need for further information is identified | 1 | 2 | 3 | 4 | 5 | 6 | 7 |
| 4. | The student demonstrates basic knowledge of the key features of common conditions | 1 | 2 | 3 | 4 | 5 | 6 | 7 |
| 5. | The student demonstrates some understanding about the department or organization where they will be undertaking the placement | 1 | 2 | 3 | 4 | 5 | 6 | 7 |
| 6. | The student demonstrates knowledge of basic treatment principles for common conditions | 1 | 2 | 3 | 4 | 5 | 6 | 7 |
| 7. | The student demonstrates knowledge of forms of treatment that may be detrimental (harmful) to a client | 1 | 2 | 3 | 4 | 5 | 6 | 7 |
| 8. | The student demonstrates knowledge of other professions and their roles | 1 | 2 | 3 | 4 | 5 | 6 | 7 |
| 9. | The student has an understanding of own learning style | 1 | 2 | 3 | 4 | 5 | 6 | 7 |
| 10. | The student demonstrates knowledge of the clinical assessment tools their educator is using to assess them | 1 | 2 | 3 | 4 | 5 | 6 | 7 |
| 11. | The student demonstrates reasoning skills | 1 | 2 | 3 | 4 | 5 | 6 | 7 |
| 12. | The student is willing to work as a team with peers, colleagues and other health professionals | 1 | 2 | 3 | 4 | 5 | 6 | 7 |
| 13. | The student is willing to ask questions and clarify to ensure understanding | 1 | 2 | 3 | 4 | 5 | 6 | 7 |
| 14. | The student is willing to try new techniques | 1 | 2 | 3 | 4 | 5 | 6 | 7 |
| 15. | The student is willing to discuss and exchange ideas to maximize patient care | 1 | 2 | 3 | 4 | 5 | 6 | 7 |
| 16. | The student is willing to receive feedback/constructive criticisms | 1 | 2 | 3 | 4 | 5 | 6 | 7 |
| 17. | The student displays a willingness to take on board any appropriate requested task | 1 | 2 | 3 | 4 | 5 | 6 | 7 |
| 18. | The student is willing to stray from their comfort zone | 1 | 2 | 3 | 4 | 5 | 6 | 7 |
| 19. | The student is willing to adhere to positive workplace culture and routines e.g. tidying up, cleaning | 1 | 2 | 3 | 4 | 5 | 6 | 7 |
| 20. | The student is willing to take responsibility for their own learning | 1 | 2 | 3 | 4 | 5 | 6 | 7 |
| 21. | The student is willing to self-evaluate | 1 | 2 | 3 | 4 | 5 | 6 | 7 |
| 22. | The student has a thorough understanding of the code of conduct and ethics for their profession | 1 | 2 | 3 | 4 | 5 | 6 | 7 |
| 23. | The student understands their role and is able to verbalise this | 1 | 2 | 3 | 4 | 5 | 6 | 7 |
| 24. | The student arrives at the placement on time | 1 | 2 | 3 | 4 | 5 | 6 | 7 |
| 25. | The student’s appearance is appropriate for the workplace and placement (e.g. dress, closed in shoes, uniform if appropriate, visible ID badge hair, fingernails, jewellery) | 1 | 2 | 3 | 4 | 5 | 6 | 7 |
| 26. | The student complies with professional matters such as confidentiality | 1 | 2 | 3 | 4 | 5 | 6 | 7 |
| 27. | The student attends each day having demonstrated appropriate follow up from previous day | 1 | 2 | 3 | 4 | 5 | 6 | 7 |
| 28. | The student makes appropriate contact with facility/educator prior to the placement commencing | 1 | 2 | 3 | 4 | 5 | 6 | 7 |
| 29. | The student is prepared for the first day having completed the appropriate pre-reading and bringing learning resources relevant for the clinical area(s) | 1 | 2 | 3 | 4 | 5 | 6 | 7 |
| 30. | The student displays ability to maintain professional boundaries with patients/patients | 1 | 2 | 3 | 4 | 5 | 6 | 7 |
| 31. | The student respectfully engages with people from a wide range of cultures and backgrounds | 1 | 2 | 3 | 4 | 5 | 6 | 7 |
| 32. | The student demonstrates effective communication and interpersonal skills (verbal, non-verbal and listening) with patients across the lifespan | 1 | 2 | 3 | 4 | 5 | 6 | 7 |
| 33. | The student is able to liaise with key stakeholders, such as organizing appointments | 1 | 2 | 3 | 4 | 5 | 6 | 7 |
| 34. | The student is able to communicate professionally with members of the multidisciplinary team | 1 | 2 | 3 | 4 | 5 | 6 | 7 |
| 35. | The student demonstrates respectful and non-judgmental communication | 1 | 2 | 3 | 4 | 5 | 6 | 7 |
| 36. | The student has the capacity to adjust their interaction style to meet the needs of the audience, whether it be colleagues, patients or others | 1 | 2 | 3 | 4 | 5 | 6 | 7 |
| 37. | The student demonstrates effective written communication skills, in charts, letters and information for patients | 1 | 2 | 3 | 4 | 5 | 6 | 7 |
| 38. | The student demonstrates enthusiasm and interest in the placement | 1 | 2 | 3 | 4 | 5 | 6 | 7 |
| 39. | The student shows initiative | 1 | 2 | 3 | 4 | 5 | 6 | 7 |
| 40. | The student is sensitive/empathetic to client's needs and concerns | 1 | 2 | 3 | 4 | 5 | 6 | 7 |
| 41. | The student has the ability to manage stress levels | 1 | 2 | 3 | 4 | 5 | 6 | 7 |
| 42. | The student demonstrates a desire to learn | 1 | 2 | 3 | 4 | 5 | 6 | 7 |
| 43. | The student demonstrates the ability to self-reflect on performance, interactions and outcomes | 1 | 2 | 3 | 4 | 5 | 6 | 7 |
| 44. | The student has self-awareness of own limitations and is honest about current level of knowledge & skills | 1 | 2 | 3 | 4 | 5 | 6 | 7 |
| 45. | The student demonstrates the ability to apply oneself | 1 | 2 | 3 | 4 | 5 | 6 | 7 |
| 46. | The student is attentive | 1 | 2 | 3 | 4 | 5 | 6 | 7 |
| 47. | The student is curious and asks questions | 1 | 2 | 3 | 4 | 5 | 6 | 7 |
| 48. | The student is proactive | 1 | 2 | 3 | 4 | 5 | 6 | 7 |
| 49. | The student is diligent | 1 | 2 | 3 | 4 | 5 | 6 | 7 |
| 50. | The student is self-directed | 1 | 2 | 3 | 4 | 5 | 6 | 7 |
| 51. | The student is helpful | 1 | 2 | 3 | 4 | 5 | 6 | 7 |
| 52. | The student is polite | 1 | 2 | 3 | 4 | 5 | 6 | 7 |
| 53. | The student is creative | 1 | 2 | 3 | 4 | 5 | 6 | 7 |
| 54. | The student is assertive | 1 | 2 | 3 | 4 | 5 | 6 | 7 |
| 55. | The student demonstrates time management skills e.g. use of a diary, to do lists | 1 | 2 | 3 | 4 | 5 | 6 | 7 |
| 56. | The student demonstrates organizational skills | 1 | 2 | 3 | 4 | 5 | 6 | 7 |
| 57. | The student has good verbal and written skills | 1 | 2 | 3 | 4 | 5 | 6 | 7 |
| 58. | The student demonstrates good observational skills | 1 | 2 | 3 | 4 | 5 | 6 | 7 |
| 59. | The student has research skills to find basic information to fill in existing knowledge gaps | 1 | 2 | 3 | 4 | 5 | 6 | 7 |
| 60. | The student has foundation skills for the area of practice | 1 | 2 | 3 | 4 | 5 | 6 | 7 |
| 61. | The student demonstrates social skills e.g. the ability to relate personably | 1 | 2 | 3 | 4 | 5 | 6 | 7 |
| 62. | The student demonstrates problem-solving skills | 1 | 2 | 3 | 4 | 5 | 6 | 7 |

Any other student attributes that are important for clinical learning:

…………………………………………………………………………………………………………………………………………………………………………………………………………………………………

…………………………………………………………………………………………………………………………………………………………………………………………………………………………………

…………………………………………………………………………………………………………………………………………………………………………………………………………………………………

…………………………………………………………………………………………………………………………………………………………………………………………………………………………………

Any other comments:

…………………………………………………………………………………………………………………………………………………………………………………………………………………………………

…………………………………………………………………………………………………………………………………………………………………………………………………………………………………

…………………………………………………………………………………………………………………………………………………………………………………………………………………………………

…………………………………………………………………………………………………………………………………………………………………………………………………………………………………
